# *Ayapana triplinervis* Essential Oil and Its Main Component Thymohydroquinone Dimethyl Ether Inhibit Zika Virus at Doses Devoid of Toxicity in Zebrafish

**DOI:** 10.3390/molecules24193447

**Published:** 2019-09-23

**Authors:** Juliano G. Haddad, Morgane Picard, Sebastien Bénard, Claire Desvignes, Philippe Desprès, Nicolas Diotel, Chaker El Kalamouni

**Affiliations:** 1 Université de la Réunion, INSERM U1187, CNRS UMR 9192, IRD UMR 249, Unité Mixte Processus Infectieux en Milieu Insulaire Tropical, Plateforme Technologique CYROI, 94791 Sainte Clotilde, La Réunion, France; juliano.haddad@univ-reunion.fr (J.G.H.); picard.morgane.noelie@gmail.com (M.P.); philippe.despres@univ-reunion.fr (P.D.); 2 Plateforme de recherche CYROI, 2 rue Maxime Rivière, 97490 Sainte Clotilde, La Réunion, France; s.benard@cyroi.fr; 3 SAS REUNION ECOEX, 2 rue Maxime rivière, 97490 Sainte Clotilde, La Réunion, France; claire.desvignes@reunionecoex.fr; 4 Université de La Réunion, INSERM, UMR 1188 Diabète athérothrombose Thérapies Réunion Océan Indien (DéTROI), 97490 Saint-Denis, de La Réunion, France

**Keywords:** *Ayapana triplinervis*, Zika virus, essential oil, antiviral activity, zebrafish, medicinal plant

## Abstract

Zika virus (ZIKV) is an emerging mosquito-borne virus of medical concern. ZIKV infection may represent a serious disease, causing neonatal microcephaly and neurological disorders. Nowadays, there is no approved antiviral against ZIKV. Several indigenous or endemic medicinal plants from Mascarene archipelago in Indian Ocean have been found able to inhibit ZIKV infection. The purpose of our study was to determine whether essential oil (EO) from Reunion Island medicinal plant *Ayapana triplinervis*, whose thymohydroquinone dimethyl ether (THQ) is the main component has the potential to prevent ZIKV infection in human cells. Virological assays were performed on human epithelial A549 cells infected with either GFP reporter ZIKV or epidemic viral strain. Zebrafish assay was employed to evaluate the acute toxicity of THQ in vivo. We showed that both EO and THQ inhibit ZIKV infection in human cells with IC_50_ values of 38 and 45 µg/mL, respectively. At the noncytotoxic concentrations, EO and THQ reduced virus progeny production by 3-log. Time-of-drug-addition assays revealed that THQ could act as viral entry inhibitor. At the antiviral effective concentration, THQ injection in zebrafish does not lead to any signs of stress and does not impact fish survival, demonstrating the absence of acute toxicity for THQ. From our data, we propose that THQ is a new potent antiviral phytocompound against ZIKV, supporting the potential use of medicinal plants from Reunion Island as a source of natural and safe antiviral substances against medically important mosquito-borne viruses.

## 1. Introduction

Zika virus, belonging to the *Flavivirus* genus of the *Flaviviridae* family, is an enveloped and single-stranded positive-sense RNA mosquito-borne virus close to dengue virus [1]. ZIKV was first isolated in the Zika forest of Uganda in 1947 [2]. The clinical syndromes of ZIKV infection are similar to those of other arbovirus infections and include, among others, fever, headache, and muscle pain. However, unlike other flavivirus, ZIKV infection has been associated with severe disorders, like Guillain–Barré syndrome and meningoencephalitis in infected adults and congenital defects in affected infants [3,4,5]. Sexual, vertical, and blood transmissions have also been reported [4,6,7]. Since then, great efforts have been carried out, but still no vaccine or specific antiviral against ZIKV is available [8,9]. The open reading frame of ZIKV genome encodes a single polyprotein, which is post-translationally processed by cellular and viral proteases to yield three structural (Capsid, C; pre-membrane, prM; and Envelope, E) and seven nonstructural (NS1, NS2A, NS2B, NS3, NS4A, NS4B and NS5) proteins [10,11,12]. The ZIKV E-protein mediates the binding of viral particles to the host receptors and the virus enters the target cells via clathrin-mediated endocytosis [12].

We recently reported several indigenous or endemic medicinal plants from Mascarene archipelago which are able to inhibit ZIKV infection through various mechanisms of action [13,14,15]. We demonstrated that *Aphloia theiformis* and *Psiloxilon mauritianum* extracts exert a virucidal effect against ZIKV by direct interactions with the virus particles as well as epigallocatechin gallate (EGCG), the main active polyphenol component from green tea [13,14,16,17]. Furthermore, *Doratoxylon apetalum* extract interfered with ZIKV internalization during viral entry into the host cells, like the flavonoid isoquercitrin (Q3G) [15,18]. As a medicinal plant recently listed in the French Pharmacopeia, *Ayapana triplinervis* (syn.: *Eupatorium ayapana* Vent; *Eupatorium triplinerve* Vahl), belonging to the *Asteraceae* family, has been traditionally used in folk medicine, among the local people of the Mascarene Islands for its sedative, stimulant, and anti-inflammatory properties [19,20]. Numerous studies demonstrated the antimicrobial, antioxidant, and antifungal activities of *A. triplinervis* [21,22,23,24]. It has been also documented that *A. triplinervis* essential oil is a rich natural source of thymohydroquinone dimethyl ether, highlighting its antimicrobial activity [25,26,27,28,29]. Several reports have shown the therapeutic roles of THQ and its derivatives as an antimicrobial phytocompounds [29,30,31,32,33]. Herein, we investigated the antiviral activity of *A. triplinervis* EO and its major compound THQ against ZIKV. We further investigated the possible mechanism of action of THQ for inhibiting ZIKV infection. Finally, we aimed at exploring the innocuity of an acute exposure of an efficient antiviral concentration of THQ in vivo. For this, we took advantage of zebrafish as a relevant model for testing the toxicity of different compounds as previously shown in other studies [34,35,36,37]. Indeed, zebrafish is an interesting model sharing a high genomic homology with humans (>70%) as well as evolutionary conserved physiological processes, making it an interesting model for drug development and toxicity analysis [38,39].

## 2. Results and Discussion

### 2.1. Noncytotoxic Doses of Ayapana triplinervis EO Inhibit ZIKV Infection in Human Cells

**Figure 1:**
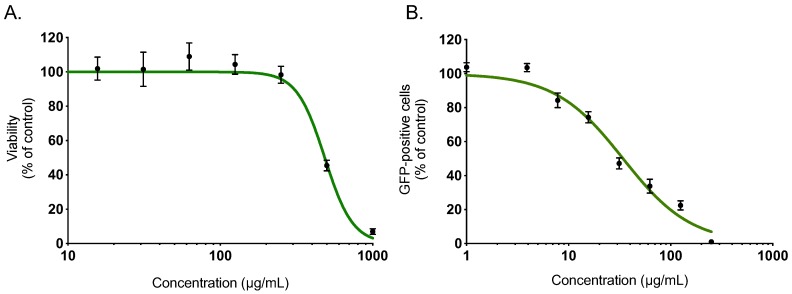
*A. triplinervis* essential oil (EO) exerts an antiviral activity against Zika virus (ZIKV). (**A**) A549 cells were incubated with two-fold serial dilutions (1000 to 7.8 µg/mL) of EO for 72 h. Cell viability was evaluated through MTT assay. Results are means ± SD of five independent experiments and are expressed as relative value compared to vehicle. (**B**) A549 cells were infected with ZIKV^GFP^ at MOI of 1 in presence of different concentrations (250, 125, 62, 5, 31, 25, 15, 62, 7, 8, 4 and 1 µg/mL) of EO. Flow cytometric analysis of GFP fluorescence was performed 24 h postinfection. The results shown are means ± SD of four independent experiments and are expressed as relative value compared to untreated infected cells.

Within a project aiming to investigate the potential antiviral activity of medicinal plants from Reunion Island recently registered in the French Pharmacopeia, this study evaluated the anti-ZIKV activity of *Ayapana triplinervis* essential oil. After extraction of EO from the fresh aerial part of *A. triplinervis*, we initially performed *A. triplinervis* EO for cellular cytotoxicity using human A549 cells. Dose-dependent experiments showed that the higher nontoxic concentration of EO (more than 90% of cell viability) was 300 µg/mL (Figure 1A). By nonlinear regression analysis, the CC_50_ value was calculated as 475 µg/mL (Table 1). We reported that ZIKV replicates efficiently in human lung epithelial A549 cells [40]. Thus, to assess whether EO inhibits ZIKV infection, A549 cells were infected 24 h with the molecular clone of ZIKV expressing the GFP (ZIKV^GFP^) at the MOI (Multiplicity Of Infection) of 1 in the presence of different concentrations (250, 125, 62,5, 31, 25, 15, 62, 7, 8, 4, and 1 µg/mL) of EO throughout the infection (Figure 1B). By flow cytometric analysis, we showed that EO treatment resulted in a severe inhibition of ZIKV infection in A549 cells (Figure 1B). The number of GFP-positive cells decreased ~80 % compared to nontreated control cells when 125 µg/mL of EO was added through the experiment (Figure 1B). The 50% inhibitory concentration (IC_50_) value was evaluated at 38 µg/mL (Table 1).

### 2.2. Thymohydroquinone Dimethyl Ether Extracted from A. triplinervis is a Potent Inhibitor of ZIKV

**Figure 2:**
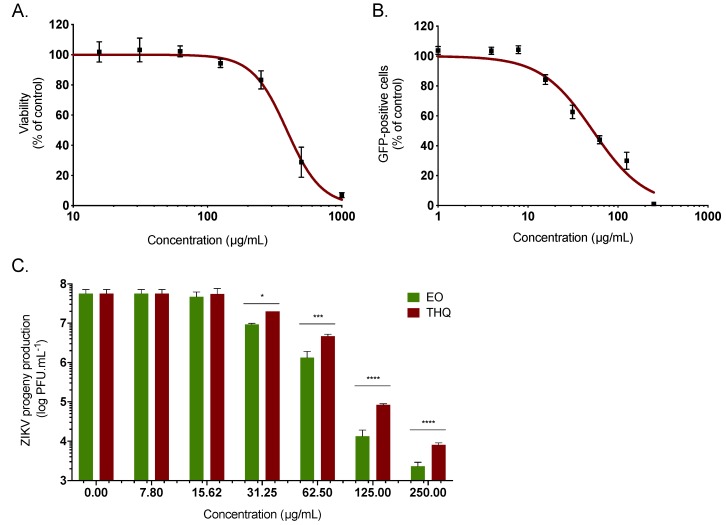
*A. triplinervis*-derived thymohydroquinone dimethyl ether (THQ) exhibits antiviral activity against ZIKV. (**A**) A549 cells were incubated with two-fold serial dilutions (1000 to 7.8 µg/mL) of THQ for 72 h. Cell viability was evaluated through MTT assay. Results are means ± SD of five independent experiments and are expressed as relative value compared to vehicle. (**B**) A549 cells were infected with ZIKV^GFP^ at MOI of 1 in presence of different concentrations (250, 125, 62, 5, 31, 25, 15, 62, 7, 8, 4, and 1 µg/mL) of THQ. Flow cytometric analysis of GFP fluorescence was performed 24 hours postinfection. The results shown are means ± SD of four independent experiments and are expressed as relative value compared to untreated infected cells. (**C**) A549 cells were infected with the cotemporary epidemic ZIKV strain (PF-25013-18) at MOI of 2 and continuously incubated with different non cytotoxic concentrations of EO or THQ. ZIKV progeny production was quantified by plaque-forming assay. Data represent the means ± SD from four independent experiments. One-way ANOVA and Dunnett’s test were used for statistical analysis (**** *p* < 0.0001; *** *p* < 0.001; * *p* < 0.05).

To identify the active components present in *A. triplinervis* EO and those responsible for the anti-ZIKV activity, the chemical composition has been identified by GC/MS analysis (Table S1). Thirty-eight volatile organic compounds were identified, accounting for 99.18% of the EO composition. The main identified compound was thymohydroquinone dimethyl ether (THQ), representing 87.06% of the total EO composition (Table S1). Such a chemical composition of *A. triplinervis* EO is consistent with a previous report for *A. triplinervis* harvested in Reunion Island, where the concentration of THQ was estimated from 89.9 to 92.8% [25]. Thus, we decided to investigate the antiviral activity of the main compound THQ isolated from *A. triplinervis* EO after purification by silica gel column chromatography and characterization by GC/MS and NMR (Figure S1). Purified THQ was first tested on A549 cells for cytotoxicity using MTT assay. THQ showed no cytotoxicity at concentrations < 150 µg/mL (Figure 2A). The CC_50_ values was calculated as 410 µg/mL (Table 1). As previously performed for EO, the antiviral activity of THQ was assessed using ZIKV molecular clone expressing the GFP. Flow cytometric analysis showed that THQ severely restricted ZIKV infection in A549 cells, yielding 80% inhibition of ZIKV infection at 125 µg/mL (Figure 2B). The IC_50_ value was 45 µg/mL (Table 1). The selectivity index (SI), which measures the preferential antiviral activity of a drug in a relation to its cytotoxicity [15], was estimated according to the values of CC_50_ and IC_50_ (SI = CC_50_/IC_50_). The SI values were 12.5 and 9.1 for EO and THQ, respectively (Table 1).

To further validate the potential anti-ZIKV activity of THQ, the cotemporary epidemic ZIKV strain (ZIKV PF-25013-18), isolated in French Polynesia during the epidemic in 2013, was used [40]. Thus, A549 cells were infected 24h at the MOI of 2 with the clinical strain in presence of increasing noncytotoxic concentrations of THQ (Figure 2C). EO was used as positive control. A dose-dependent effect of THQ on ZIKV growth was observed (Figure 2C). At the noncytotoxic concentration of 125 µg/mL, EO and THQ reduced the progeny virus production by 4- and 3-log, respectively (Figure 2C). Taken together, these data demonstrated that THQ, the main compound isolated from *A. triplinervis* EO, exerts antiviral activity against ZIKV.

### 2.3. Thymohydroquinone Dimethyl Ether Prevents ZIKV Entry in Human Cells

**Figure 3:**
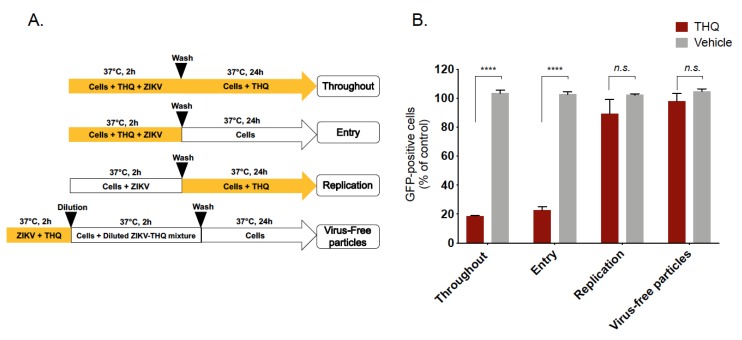
Thymohydroquinone dimethyl ether interferes with the early stage of ZIKV infection. (**A**) Schematic representation for time-of-drug-addition assay used to characterize anti-ZIKV activity of THQ (125 µg/mL) in A549 cells. Yellow arrows indicate the presence of THQ. (**B**) Results of GFP expression in ZIKV-infected A549 cells, under different conditions shown in panel A, are analyzed by cytometry assay. The results represent the mean ± SD of four independent experiments and are expressed as relative values compared to vehicle. One-way ANOVA and Dunnett’s test were used for statistical analysis (**** *p* < 0.0001; *n.s* = not significant).

To characterize the antiviral activity of THQ against ZIKV, different experimental approaches were conducted (Figure 3A). To assess the effects on the viral entry stage, ZIKV and THQ were simultaneously co-added to the cells during 2 h (Figure 3A, Entry). To investigate whether THQ interferes with ZIKV replication, A549 cells were first infected with ZIKV for 2 h and then treated with THQ (Figure 3A, Replication). To investigate whether EO directly affects the cell-free virions to abolish the subsequent infection (virucidal effect), ZIKV particles were pre-incubated with THQ for 2h, and then diluted 50-fold prior infection (Figure 3A, virus-free particles). THQ was also maintained throughout the experimental period as positive control condition (Figure 3A, throughout). The preincubation of ZIKV with THQ prior to incubation with A549 cells demonstrated that THQ does not exhibit virucidal effect against ZIKV (Figure 3B). Also, the treatment of cells with THQ showed no significant effect on ZIKV replication. The results shown in Figure 3B suggest that THQ-mediated inhibition was associated to an inability of ZIKV to initiate a productive infectious cycle. All together, these data suggest that THQ targets the initial stages of ZIKV infection.

**Figure 4:**
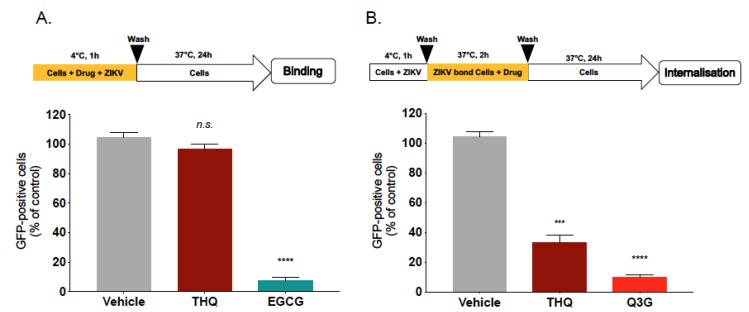
THQ interferes with the internalization step of ZIKV entry. (**A**) A549 cells were infected with ZIKV^GFP^ at MOI of 1 for 1 h at 4°C in the presence of THQ (125 µg/mL), and then the temperature was shifted to 37 °C in absence of THQ. Epigallocatechin gallate, EGCG (100 µM), was used as positive control. (**B**) A549 cells were infected for 1h with ZIKV^GFP^ at 4 °C, then the temperature was shifted to 37°C in presence of THQ. Isoquercitrin, Q3G (100 µM), was used as positive control. Flow cytometric analysis of GFP fluorescence was performed 24 h postinfection. The results shown are means ± SD of four independent experiments and are expressed as relative value compared to untreated infected cells. One-way ANOVA and Dunnett’s test were used for statistical analysis (*** *p* < 0.001; **** *p* < 0.0001; *n.s* = not significant).

To further elucidate the underlying mechanism of THQ antiviral activity, we subsequently tested the effect of THQ on ZIKV entry steps. We first investigated whether THQ was able to affect viral attachment. Thus, binding assays were performed at 4 °C which allows virus binding but prevents viral entry (Figure 4A). Epigallocatechin gallate, EGCG, the main polyphenol compound from green tea, which is known to inhibit ZIKV binding, was used as positive control [14,16,17]. After 1h of ZIKV attachment in the presence of THQ, the temperature was shifted to 37 °C without THQ to allow ZIKV internalization (Figure 4A). Our data show that the percentage of fluorescence in ZIKV-infected A549 cells was similar to untreated control cells. However, EGCG significantly inhibits ZIKV attachment to the cell membrane (Figure 4A). Taken together, these results demonstrate that THQ is not able to inhibit ZIKV binding, suggesting that a postattachment step of the infectious virus cycle could be altered.

To investigate whether THQ inhibits ZIKV internalization step, A549 cells were infected with ZIKV^GFP^ at 4 °C for 1h without THQ, then the temperature was shifted to 37 °C to allow virus penetration, in the presence of THQ (Figure 4B). The natural flavonoid Q3G, which is known to inhibit ZIKV internalization, was used as positive control [18]. Flow cytometric analysis showed that the percentage of ZIKV-infected A549 cells was severely reduced in presence of THQ, as well as the positive control Q3G (Figure 4B). These results suggest that THQ is a potent inhibitor of ZIKV internalization process. Thus, THQ-mediated inhibition of ZIKV infection occurs early after virus binding to the cell membrane and could be explained by the incapacity of the ZIKV-attached particles to be internalized into the host cell in presence of THQ.

### 2.4. The Anti-ZIKV Inhibitor Dose of Thymohydroquinone Dimethyl Ether Causes no Toxic Effect in a Zebrafish Model

**Figure 5:**
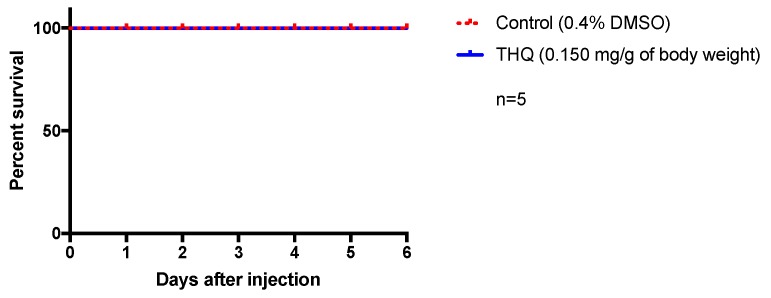
Acute exposure of THQ does not induce apparent toxicity in fish. Control fish were injected with vehicle (0.4% DMSO) and treated ones were injected with THQ from *A triplinervis* EO. After injection, fish were observed during 6 days. THQ-treated fish showed normal feeding and locomotor behaviors, and did not display any striking sign of stress. Their survival was not affected by the treatment and was similar to control. n = number of fish per group.

To investigate the innocuity of the antiviral dose of THQ in vivo, we decided to use zebrafish as a relevant physiological model. Indeed, zebrafish shares a high genomic homology with humans (>70%) as well as many physiological processes, making it an interesting model for toxicity test and drug development [38,39]. We consequently performed intraperitoneal injection of THQ (0.15 mg/g of body weight) for evaluating its potential acute toxicity. This dose (0.15 mg/g of body weight) was chosen as it corresponds to the maximum noncytotoxic concentration estimated in vitro. Any sign of suffering and behavioral changes were carefully checked every day and fish survival was monitored for 6 days. Interestingly, no striking behavioral changes (locomotor activity, feeding behavior, and stress) have been detected in THQ-treated fish compared to controls. In addition, as shown by the respective Kaplan–Meier curves, from the injection to day 6, THQ-injected fish display a 100% survival similar to the control (Figure 5). Taken together, these data suggest that THQ does not exhibit acute toxicity in vivo in our experimental conditions.

### 2.5. Concluding Remarks

Recently, we reported that a nonvolatile extract isolated from *A. triplinervis* in Reunion Island exhibited no antiviral effect against ZIKV [14]. In the present study, we showed that a volatile extract from the same plant and represented by an essential oil was efficacious against ZIKV. Whereas the nonvolatile fraction from *A. triplinervis*, which is mainly constituted of alkaloids, coumarins, and polyphenols, has no antiviral effect [23,41]. The terpene derivative-rich *A. triplinervis* EO was effective against ZIKV. We found that a major phytocompound in *A. triplinervis* EO, the thymohydroquinone dimethyl ether, accounts for inhibition of ZIKV in human cells. Interestingly, both EO and THQ are effective against the contemporary epidemic ZIKV strain reducing ZIKV growth up to 3-log. To our knowledge, this is the first time that a noncytotoxic dose of EO and THQ has been demonstrated efficacious against a mosquito-borne RNA virus of medical concern.

Early viral entry assays revealed that THQ-mediated ZIKV inhibition relates to a blockade in the early stages of virus infection presumably at viral entry level. The antiviral mechanism of THQ is likely close to that of natural flavonoid Q3G as it has been reported elsewhere [18]. Indeed, we hypothesized that THQ-mediated ZIKV inhibition is due to a defect in the endocytosis steps of virus particles. It has recently been reported that clathrin-mediated endocytosis pathway involving Axl/Gas6 as entry factors may play a key role in ZIKV entry into the host-cell [42]. Whether THQ-mediated ZIKV inhibition involves Axl/Gas6 entry factors remains to be investigated. Such studies would also broaden our knowledge on the antiviral mechanisms associated to phytocompounds, such as THQ.

In conclusion, our data identified, for the first time, the medicinal plant *A. triplinervis* as a new source of antiviral phytocompounds targeting the cell entry of viral pathogens [13,14,15,18,43,44]. We demonstrated that THQ from *A. triplinervis* EO is a potent inhibitor of ZIKV infection in human cells. It is of great interest to evaluate the toxicity of *A. triplinervis* major phytocompound THQ in vivo. Consequently, we took advantage of a newly established zebrafish model to test the biosafety of THQ extracted from *A. triplinervis* [35,37,38]. We noted that inoculation of THQ at the doses showing antiviral effect against ZIKV in vitro caused no obvious stress nor impaired locomotor and feeding behaviors in inoculated zebrafish. The data obtained in a zebrafish experimental model reinforce the great interest that represents THQ as a newly identified antiviral phytocompound, which could be potentially efficacious against different enveloped RNA viruses known for their incidence in terms of public health. The results presented in this study underscore viral entry inhibitors as a promising class of antivirals and add *A. triplinervis* to the group of medicinal plants and their phytochemicals that interfere with the early stage of ZIKV replication cycle. Whether or not EO and THQ also exert antiviral activity against other medically important flaviviruses such as dengue virus, yellow fever virus, and West Nile virus is a critical issue that remains to be investigated.

## 3. Materials and Methods

### 3.1. Cells, Viruses and Reagents

Human lung epithelial A549 cells (ATCC, CCL-185, Manassas, VA, USA) and Vero cells (ATCC, CCL-81) were grown in minimum essential medium (MEM: Gibco/Invitrogen, Carlsbad, CA, USA) supplemented with 10% heat-inactivated fetal bovine serum (FBS Good: Invitrogen), 2 mmol·L^−1^ L-Glutamine, 1 mmol·L^−1^ sodium pyruvate, 100 U·mL^−1^ of penicillin, 0.1 mg·mL^−1^ of streptomycin, and 0.5 µg·mL^−1^ of fungizone (PAN Biotech) under a 5% CO_2_ atmosphere at 37 °C. The clinical isolate PF-25013-18 of ZIKV (ZIKV-PF13) and the recombinant Zika virus expressing the GFP reporter gene (ZIKV^GFP^) have been previously described [40,45,46]. The ZIKV progeny production was determined by measuring the quantity of infectious particles released into the supernatant by plaque-forming assay on Vero cells as previously described [13,15]. EGCG and Q3G were purchased from Sigma-Aldrich. The growth culture medium supplemented with 0.4% of DMSO was used as a vehicle control.

### 3.2. Extraction and Chemical Characterization of Essential Oil

The fresh aerial parts of *A. triplinervis* harvested in the East of Reunion Island were subjected to steam distillation assisted with a turbomixer. After 1h, the EO was collected and dried on Na_2_SO_4_ and stored in a dark-sealed vial until analysis.

GC analysis was performed on a ThermoFisher gas chromatograph (Trace 1300), equipped with a flame ionization detector. A ZB-5MS capillary column (30 mm, 0.25mm coated with 5% pheny-Arylene and 95% dimethyl polysiloxane, and 0.25 µm film thickness) and helium as the carrier gas (1 mL/min) were used. Percentages of compounds were determined from their peak areas in the GC-FID profiles. GC–MS analysis was performed on the above instrument, coupled with a triple quadrupole mass detector GC–MS/MS (Thermo Scientific TSQ 8000 Evo) with the same column and the same operative conditions used for the analytical GC. The ionization voltage was 70 eV, the ion source temperature was 250 °C. Detected compounds were identified based on the following parameters: GC retention index (relative to C8–C23 n-alkanes), values reported in the literature [25,26], matching of mass spectral data with those of the MS libraries (NIST14, Wiley11, FFNSC3 and Adams [47]).

### 3.3. Purification and Characterization of Thymohydroquinone Dimethyl Ether

Thymohydroquinone dimethyl ether was isolated from a sample of *A. triplinervis* essential oil by flash column chromatography (silica gel). Hexane was used as a mobile phase to afford the desired compound as a colorless oil. Thymohydroquinone dimethyl ether was characterized by ^1^H NMR spectroscopy and mass spectroscopy [30]. Thymohydroquinone dimethyl ether : Rf: 0.26 (SiO_2_, Hexane), ^1^H NMR (CDCl_3_, 600 MHz) : 1.21 (d, *J* = 7.0 Hz, 6H, 2XCH_3_ (-iPr)), 2.21 (s, 3H, Ar-Me), 3.30 (hept, J = 7.0 Hz, 1H, -CH- (iPr)), 3.79 (s, 3H, -OMe), 3.81 (s, 3H, -OMe), 6.68 (s,1H, Ar-H), 6.73 (s,1H, Ar-H). MS (EI): 194.10, 179.08, 164.07.

### 3.4. MTT Assay

Cell viability was measured using the tetrazolium salt MTT assay as described previously [14,18]. Briefly, A549 cells were treated 72 h at 37 °C with EO or THQ within a wide range of concentrations (38–1235 µg/mL). The essential oils and THQ were solubilized in 0.4% DMSO. Following the treatment, 20 µL of 5 mg/ml MTT solution (Sigma) were added to the 96-well plates, and cells were stored at 37 °C in the darkness. After 3 h of treatment, supernatant was removed and replaced by 100 µL of DMSO. The MTT medium was removed, and the formazan crystals were solubilized with 50 µL of DMSO. Absorbance was measured at 570 nm with a background subtraction at 690 nm. The CC_50_ was determined using a nonlinear regression on Graphpad prism software.

### 3.5. Flow Cytometry Assay

For cytometry assay, cells were harvested, fixed with 3.7% PFA in PBS for 20 min, washed twice with PBS, and then subjected to a flow cytometric analysis using Cytoflex (Beckman). Results were analyzed using cytExpert software.

### 3.6. Virus Inactivation Assay

To measure the direct effect of the EO and THQ on viral infectivity (free-virus particles), ZIKV^GFP^ (2 × 10^5^ PFU) were mixed with EO or THQ at 125 µg/mL and then incubated at 37 °C for 2 h. The mixture was diluted 50-fold (final virus concentration, 1 PFU/cell) in MEM containing 10% FBS to yield a subtherapeutic concentration of EO or THQ, and this mixture was subsequently added to A549 cells monolayer seeded in a 6-well plates. As a comparison, ZIKV^GFP^ was mixed with EO or THQ, diluted immediately to 50-fold (no incubation period), and added to cells for infection. After 2 h of adsorption at 37 °C, the diluted inocula were discarded, and cells were washed with PBS twice. Medium overlay was applied and the plates were further incubated at 37 °C for 24 h.

### 3.7. Evaluation of Toxicity In Vivo

Three- to six-month-old male adult wild type zebrafish (Danio rerio) were maintained under standard conditions of temperature (28 °C), photoperiod (14/10 hr light–dark), and conductivity (400 μS). Zebrafish were fed daily with Gemma Micro ZF 300 (Planktovie). For intraperitoneal injections, fish were deeply anesthetized with 0.02% tricaine (MS-222; REF: A5040, Sigma-Aldrich) and injected with vehicle (PBS—0.4% DMSO), and the main purified compound thymohydroquinone dimethyl ether extracted from *Ayapana triplinervis* EO (THQ, 0.150 mg/g). This dose was chosen as it corresponds to the maximum noncytotoxic concentration that has been tested in vitro, assuming that 1 gram of zebrafish is closed to 1 mL volume. Animals were carefully checked for stress, locomotor activity, and feeding behavior. All animal experiments were conducted in accordance with the French and European Community Guidelines for the Use of Animals in Research (86/609/EEC and 2010/63/EU) and approved by the local Ethics Committee for animal experimentation of CYROI (APAFIS #2018072310507016_v5). At the end of the procedure, the animals were sacrificed with overdose of tricaine.

### 3.8. Data Analysis

A comparison between the different concentrations was done by a one-way ANOVA test. All values were expressed as mean ± SD of at least three independent experiments. All statistical tests were done using the software Graph-Pad Prism (version 8.0; GraphPad software, La Jola, CA, USA). Degrees of significance are indicated on the figure as follows; * *p* < 0.05; ** *p* < 0.01; *** *p* < 0.001, **** *p* < 0.0001, *n.s* = not significant.

## Figures and Tables

**Table 1:** Cytotoxicity and antiviral activity of EO and THQ.

Compound	CC_50_ (µg/mL) ^a^	IC_50_ (µg/mL) ^b^	SI ^c^
EO	475 ± 48	38 ± 4.2	12.5
THQ	410 ± 46	45 ± 7.5	9.1

Cytotoxic concentration (CC_50_) and inhibitory concentration (IC_50_) were obtained by performing nonlinear regression followed by the construction of the sigmoidal concentration–response curves from Figure 1 and Figure 2A,B. ^a^ Concentration that inhibited cell viability by 50%; ^b^ Concentration that inhibited infection by 50%; ^c^ Selectivity index (CC_50_/IC_50_).

## Supplementary Materials

The following are available online. Table S1: Chemical composition of *Ayapana triplinervis* essential oil (aerial part) from Reunion Island; Figure S1: Characterization of thymohydroquinone dimethyl ether after purification from *A. triplinervis* essential oil.
