# Supplementary material for: Ayapana triplinervis Essential Oil and Its Main Component Thymohydroquinone Dimethyl Ether Inhibit Zika Virus at Doses Devoid of Toxicity in Zebrafish
Source: Molecules. 2019 Sep 23;24(19):3447. doi: 10.3390/molecules24193447 (PMC6804133; doi:10.3390/molecules24193447)
Supplement: Supplementary file 1 [file molecules-24-03447-s001.pdf]

Table S1. Chemical composition of *Ayapana triplinervis* essential oil (aerial part) from Reunion Island

No	Compounds	KI[a]	Identification	Composition (%)
1	Tricyclene	923	KI, MS	< 0.01
2	α-Thujene	931	KI, MS	0.99
3	β-Pinene	976	KI, MS	1.82
4	Myrcene	985	KI, MS	0.06
5	α-Phellandrene	1006	KI, MS	2.03
6	*p*-Cymene	1022	KI, MS	0.68
7	Limonene	1026	KI, MS	0.22
8	β-Phellandrene	1028	KI, MS	< 0.01
9	(Z)-β-Ocimene	1041	KI, MS	0.02
10	(E)- 4,8-dimethyl-,1,3,7-nonatriene	1110	KI, MS	0.15
11	Thymol methyl ether *isomer	1225	KI, MS	1.46
12	Thymol methyl ether	1234	KI, MS	0.36
13	Carvacrol methyl ether	1245	KI, MS	< 0.01
14	(E)-Sabinyl acetate	1291	KI, MS	0.07
15	Neryl acetate	1364	KI, MS	0.04
16	Linalool isobutyrate	1379	KI, MS	< 0.01
17	β-Elemene	1384	KI, MS	0.09
18	Cyperene	1397	KI, MS	0.08
19	**Thymohydroquinone dimethyl ether**	**1420**	**KI, MS**	**87.06**
20	1,4-Dimethoxy-2-methyl-5-(prop-1-en-2-yl) benzene	1432	KI, MS	0.04
21	α-Humulene	1452	KI, MS	0.03
22	Drima-7,9(11)-diene	1465	KI, MS	< 0.01
23	β-Chamigrene	1468	KI, MS	0.07
24	β-Selinene	1483	KI, MS	1.87
25	Bicyclogermacrene	1489	KI, MS	< 0.01
26	α-Muurolene	1500	KI, MS	0.20
27	δ-Amorphene	1511	KI, MS	0.03
28	β-Sesquiphellandrene	1518	KI, MS	< 0.01
29	Elemol	1541	KI, MS	0.03
30	Tert-Butyl 2-(4-methoxyphenyl) acetate	1550	KI, MS	< 0.01
31	(E)-Nerolidol	1555	KI, MS	0.03
32	Caryophyllene oxide	1573	KI, MS	0.07
33	5-Cedranone *isomer	1609	KI, MS	0.74
34	5-Cedranone	1612	KI, MS	0.20
35	α-Eudesmol	1649	KI, MS	0.08
36	Ar Turmerone	1656	KI, MS	0.32
37	Ar-Turmerone *isomer	1661	KI, MS	0.23
38	β-Turmerone	1693	KI, MS	0.11

[a] Kováts retention indices calculated against C_8–C_{23} *n*-alkanes on nonpolar ZB-5MS column.

A.

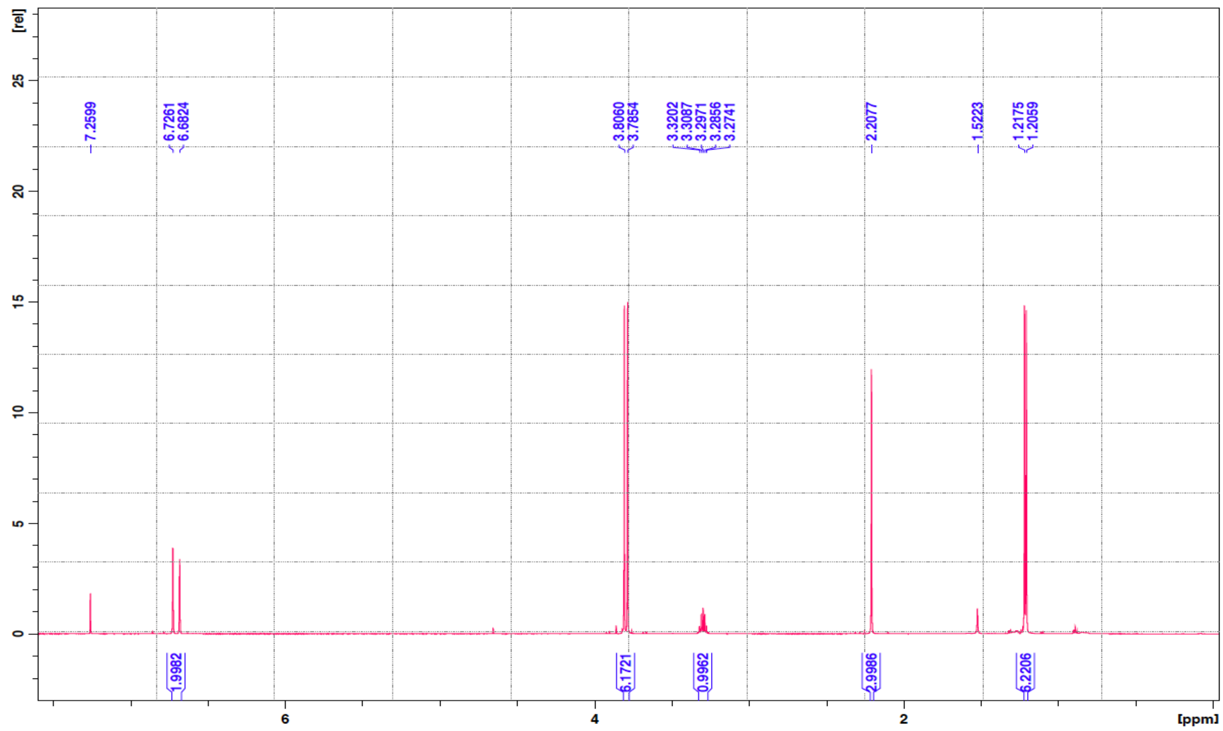

B.

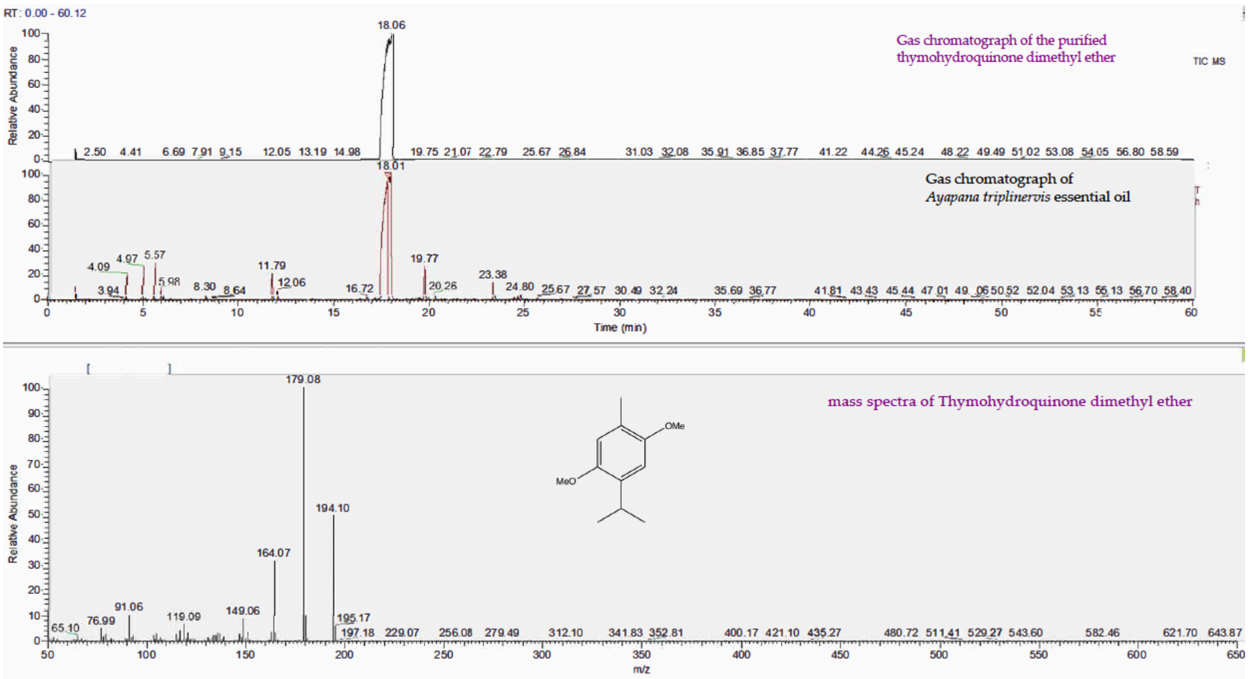

Figure S1. Characterisation of thymohydroquinone dimethyl ether after purification from *A. triplinervis* essential oil. (A). ^{1}H NMR spectra of purified thymohydroquinone dimethyl ether. **(B).** Gas chromatograph of purified thymohydroquinone dimethyl ether and *A. triplinervis* essential oil.
